# Association of −31T>C and −511 C>T polymorphisms in the interleukin 1 beta (*IL1B*) promoter in Korean keratoconus patients

**Published:** 2008-11-21

**Authors:** So-Hee Kim, Jee-Won Mok, Hyun-Seok Kim, C.K. Joo

**Affiliations:** 1 Department of Life Science, Catholic University of Korea, Seoul, Korea; 2 Laboratory of Ophthalmology and Visual Science, Catholic University of Korea, Seoul, Korea; 3 Korea Eye Tissue and Gene Bank, Catholic University of Korea, Seoul, Korea; 4 Department of Ophthalmology, St. Mary’s Hospital, Seoul, Korea

## Abstract

**Purpose:**

To investigate the genetic association between unrelated Korean keratoconus patients and interleukin 1 alpha (*IL1A*), interleukin 1 beta (*IL1B*), and IL1 receptor antagonist (*IL1RN)* gene polymorphisms.

**Methods:**

We investigated the association between *IL1A* (rs1800587, rs2071376, and rs17561), *IL1B* (rs1143627, rs16944, rs1143634, and rs1143633), and *IL1RN* (rs419598, rs423904, rs424078, and rs315952, variable number tandem repeat [VNTR]) polymorphisms in 100 unrelated Korean keratoconus patients. One hundred control individuals without any corneal disease were selected from the general population. Polymerase chain reaction (PCR) – restriction fragment length polymorphism (RFLP) analysis and direct sequencing were used to screen for genetic variations in the *IL1* gene cluster. Haplotypes for the *IL1* gene cluster were constructed using Haploview version 4.0.

**Results:**

We analyzed a total of 12 polymorphic sites in the *IL1* gene cluster. Among them, the −511 (rs16944) and −31 (rs1143627) positions in the promoter region of *IL1B* were significantly different between patient and control groups. The C allele of rs16944 (−511C>T, p=0.022, odds ratio of risk [OR]=1.46, 95% confidence intervals [CI] 0.94<2.27) and the T allele of rs1143627 (−31T>C, p=0.025, OR=1.43, 95% CI 0.92<2.22) were associated with a significantly increased risk of keratoconus in Korean patients. Linkage of the two alleles, −31*C and −511*T, was associated with an increased risk for keratoconus with OR=2.38 (p=0.012, 95% CI=1.116–5.046). The *C/*A genotype of rs2071376 in *IL1A* intron 6 was significantly different between the keratoconus patients and control subjects (p=0.034, OR=0.59, 95% CI 0.32<1.11). Other polymorphisms did not show an association with keratoconus risk.

**Conclusions:**

This is the first report of *IL1* gene cluster mutation screening in Korean keratoconus patients. Significant differences in allelic frequency of *IL1B* between keratoconus patients and the control group suggest that *IL1B* polymorphisms may play a role in the susceptibility of unrelated Koreans to develop keratoconus.

## Introduction

Keratoconus (OMIM 148300) is a bilateral, asymmetric, chronic, progressive ectasia of the cornea characterized by the steepening and distortion of the cornea, thinning of the apical cornea, and corneal scarring, which leads to progressive myopic and irregular astigmatism [1-3]. Histologically, the keratoconic cornea stroma may become less than one-quarter its normal thickness thereby leading to extensive distortion [4]. The pathophysiological processes underlying the keratoconic cornea have yet to be fully elucidated, although various studies have suggested that keratoconus is associated with eye rubbing in atopic patients [2,5], contact lens wearing [6], increased proteinase activity [4,7], decreased levels of proteinase inhibitors [8-10], increased oxidative damage [11], and keratocyte apoptosis [12,13].

Keratocyte apoptosis has been reported in 60% of keratoconic corneas and is triggered by the epithelial release of interleukin 1 (IL1), which is activated by chronic mechanical injury to the corneal epithelium [12,14]. More specifically, IL1 has been implicated as a damage mediator, which is a modulator that regulates the apoptotic process [15,16], in these eye rubbers. In addition, stromal thinning in keratoconus is caused by the stimulation of keratocyte apoptosis induced by IL1. For these reasons, *IL1* has been suggested as a candidate gene for keratoconus.

IL1 is a pleiotropic cytokine. It is involved in the inflammatory response, cell growth, and tissue repair in the cortex. The IL1 superfamily consists of three members, IL1 alpha (IL1α), IL1 beta (IL1β, predominant form) and IL1 receptor antagonist (IL1Ra), which are encoded by *IL1A*, *IL1B*, and *IL1RN*, respectively [17,18]. The *IL1* superfamily genes are located in tandem in a cluster on chromosome 2q14. This gene cluster contains several polymorphisms [19]. Among them, some polymorphisms are located within the regulatory regions of the genes. Their localization in regulatory regions suggests that they may modulate IL1 protein production by directly affecting transcription, leading to their association with altered levels of IL1 [18,20-22].

The goal of this study was to elucidate whether polymorphisms in *IL1A*, *IL1B*, and *IL1RN* are associated with keratoconus in Korean patients. We determined the genotype frequencies of 11 single nucleotide polymorphisms (SNPs), which were associated with altered levels of cytokines, and one variable number tandem repeat (VNTR) marker in the *IL1* gene cluster in unrelated Korean keratoconus patients. Identification of genetic factors that determine susceptibility to keratoconus in Korean patients may allow us to gain insight into the pathogenesis of keratoconus.

## Methods

This study included 100 unrelated keratoconus patients of Korean descent with age ranging from 18 to 33 years old. All of the keratoconus patients were identified from the Korea Eye Tissue and Gene Bank related to Blindness in the Department of Ophthalmology at the Catholic University of Korea (Seoul, Korea). Appropriate informed consent was obtained from each subject, and all studies were performed according to the tenets of the Declaration of Helsinki. The patients were diagnosed with keratoconus based on the following criteria: (1) symptoms of keratoconus including the Munson sign, protrusion, Vogt’s striae, corneal thickness, scarring, the Fleischer ring, signs of photokeratoscopy, signs of videokeratography, and refractive errors and (2) medical histories including age, sex, contact lens use, eye rubbing behavior, systemic disease, atopy, and connective tissue disease [2,23]. One hundred age-matched control individuals with no history of keratoconus were also enrolled from the Korea Eye Tissue and Gene Bank related to Blindness.

DNA was extracted from peripheral blood samples using the QIAamp DNA blood kit (QIAGEN, Valencia, CA). Polymerase chain reaction (PCR) reactions were performed with 25 ng of genomic DNA as a template in a mixture of PCR buffer, 2.5 mM MgCl_2_, 200 nM dNTPs, 0.4 pmol of each primer, and 0.75 units of h-Taq polymerase (Solgent, Daejeon, Korea; Table 1) [19,24,25].

**Table 1:** Polymorphic sites of *IL1A*, *IL1B,* and *IL1RN*.

	**Nucleotide**	**SNPs**	**dbSNPs**	**Amino acid**	**Assay method^a^**	**Genotypes^b^**	
*IL1A*						
exon 1	−889 C>T	Ex1+12C>T	rs1800587		RFLP: DpnII	*c: 170bp	*t: 145+25bp
exon 5	+4845 G>T	Ex5+21G>T	rs17561	A114S	RFLP: SatI	*G:29+123+3+84bp	*T:152+3+84bp
intron 6	+376 C>A	IVS6+169C>A	rs2071376		RFLP: BstYI	*c:270bp	*a: 198+72bp
*IL1B*						
promoter	−511C>T	−511T>C	rs16944		RFLP: AvaI	*t:530bp	*c:190+340bp
promoter	−31 T>C	−31 C>T	rs1143627		Direct sequencing		
intron 4	+5810 G>A	IVS4–64G>A	rs1143633		RFLP: Fnu4HI	*g:38+271+19bp	*a:309+19bp
exon 5	+3954C>T	Ex5+^14^C>T	rs1143634	F105F	RFLP: TaqI	*C:116+212bp	*T:328bp
*IL1RN*						
exon 4	+8006 C>T	EX4+55C>T	rs419598	A60A	RFLP: HpaII	*C: 29 +98 bp	*T: 127 bp
intron 4	+8061 C>T	IVS4+21C>T	rs423904		RFLP: MwoI	*c:179+46bp	*t:225bp
intron 4	86bp VNTR				Agarose	I: 410 bp, II: 240bp, III: 500bp, IV: 325 bp, V: 595 bp	
intron 5	+9589A>T	IVS5+59A>T	rs454078		RFLP: SspI	*a:299bp	*t:145+154bp
exon 7	+11100T>C	Ex7+72T>C	rs315952	S133S	RFLP: MspA1I	*T:330bp	*C:132+198bp

Assay method^a^: Screening method for polymorphic site and lists of restriction enzyme; Agarose: Agarose gel electrophoresis; Genotypes^b^: It was indicated restricted sizes by RFLP analysis and amplified products for VNTR

Biallelic polymorphisms in the *IL1* gene cluster were determined by PCR–restriction fragment length polymorphism (RFLP) analysis and direct sequencing (Table 1). The *IL1RN* VNTR polymorphism was evaluated by identifying the number of repeats in the (86)n sequence using agarose gel electrophoresis and direct sequencing [19,24,25].

The Hardy–Weinberg equilibrium (HWE) was calculated using the GenePop web version 4.0 program. To determine statistically significant differences in genotype and allele frequencies between the two groups, we used the χ^2^ test or Fisher’s exact test for the 2×2 contingency table file. The descriptive statistics for observed differences in allele or genotype distribution with the corresponding p values were analyzed using the JavaStat web software in combination with StatXact-8 software (Cytel Inc., Cambridge, MA). The strength of the association was estimated by odds ratio of risk (OR) and 95% confidence intervals (CI). Haplotype frequencies and associations were calculated with Haploview (version 4.0) that uses the expectation maximization (EM) algorithm [26]. Haplotype distributions were evaluated by the permutation test on the basis of 10,000 replications to obtain the empirical significance [26]. Values of p<0.05 were considered statistically significant.

## Results

**Figure 1:**
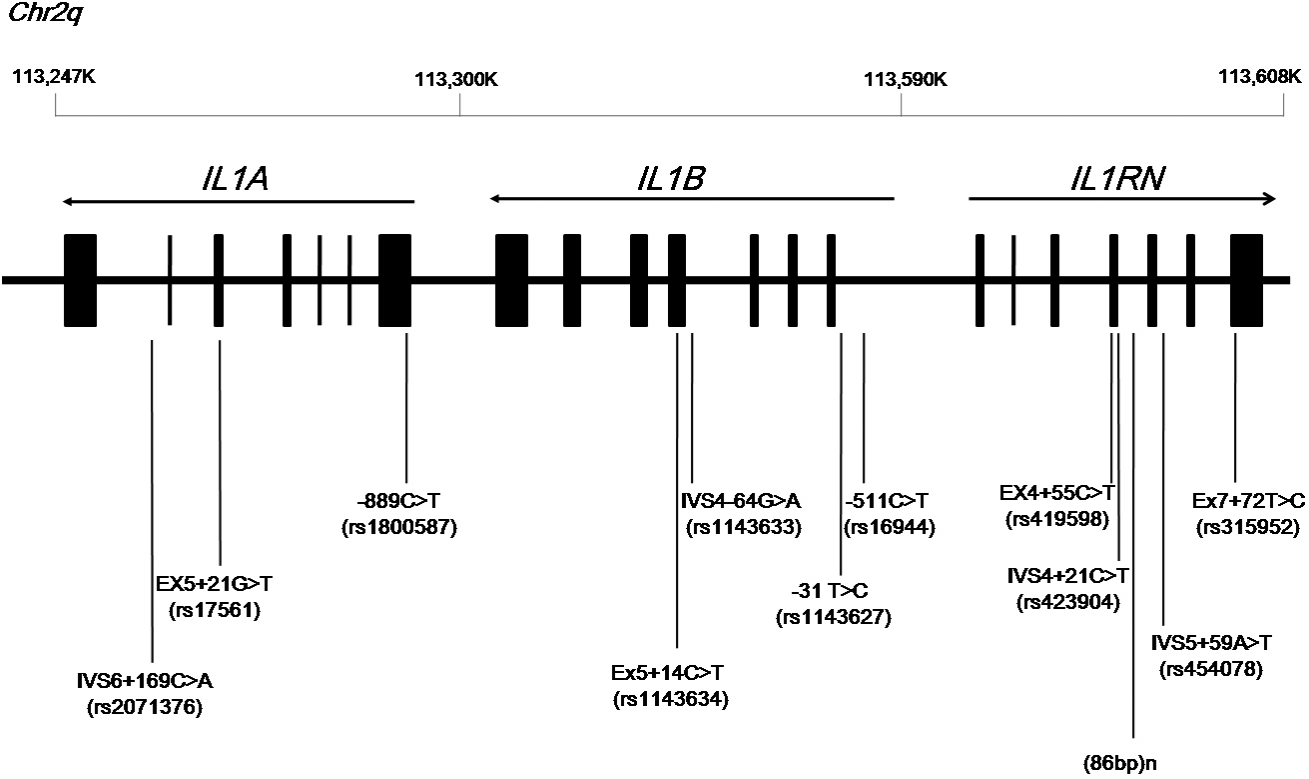
*IL1A*, *IL1B*, and *IL1RN*, showing polymorphic nucleotide sequences. The map of the three genes, which are located on 2q14, is according to the UCSC Human Genome Browser Mar 2006 Assembly. The direction of transcription is shown above each gene by a horizontal arrow. Exons are shown as rectangles. Vertical lines indicate polymorphic sites evaluated in this study.

We analyzed 11 SNPs and one VNTR marker in the *IL1* gene cluster: rs1800587, rs2071376, and rs17561 for *IL1A*; rs1143627, rs16944, rs1143634, and rs1143633 for *IL1B*; and rs419598, rs423904, rs424078, rs315952, and the (86)n VNTR marker for *IL1RN* (Figure 1). The *IL1A*, *IL1B*, and *IL1RN* genotypic and allelic frequencies in keratoconus patients are listed in Table 2 and Table 3. The genotype distributions of the variants of the 12 polymorphisms in the *IL1* gene cluster among the control subjects and the keratoconus patients were in Hardy–Weinberg equilibrium.

**Table 2:** Genotype frequencies of *IL1A* and *IL1B* in keratoconus patients.

**Genes**	**SNPs**	**Genotypes**	**Frequency (%)**		**p value**
			**Keratoconus**	**Control**	
*IL1A*	−889 C>T (rs1800587)	C/C	87.2	88.6	N.S.
		T/C	11.7	10	N.S.
		T/T	1.1	1.4	N.S.
	+4845 G>T (rs17561)	G/G	75.8	81.4	N.S.
		T/G	23.2	17.1	N.S.
		T/T	1.1	1.4	N.S.
	+376 C>A (rs2071376)	A/A	11.7	8.6	N.S.
		C/A	37.2	50	0.034*
		C/C	51.1	41.4	N.S.
*IL1B*	−511C>T (rs16944)	C/C	34.8	21.4	0.025**
		T/C	43.5	51.5	N.S.
		T/T	21.7	27.1	N.S.
	−31 T>C (rs1143627)	C/C	23.7	28.6	N.S.
		T/C	41.9	50	N.S.
		T/T	34.4	21.4	0.027***
	+5810G>A (rs1143633)	A/A	39.8	45.7	N.S.
		G/A	39.8	35.7	N.S.
		G/G	20.4	18.6	N.S.
	+3954C>T (rs1143634)	C/C	92.5	95.7	N.S.
		T/C	6.5	2.9	N.S.
		T/T	1.1	1.4	N.S.

The asterisk indicates that OR=0.59 and 95% CI=0.32<1.11. The double asterisk indicates that OR=1.96 and 95% CI=0.96<3.96. The triple asterisk indicates that OR=1.92 and 95% CI=0.95<3.90. N.S., not significant.

**Table 3:** Genotype frequencies of *IL1RN* in keratoconus patients.

**Gene**	**SNP**	**Genotypes**	**Frequency (%)**		**p value**
			**Keratoconus**	**Control**	
*IL1RN*	+8006 C>T (rs419598)	C/C	2.1	0.0	N.S.
		T/C	11.6	12.9	N.S.
		T/T	86.3	87.1	N.S.
	+8061 C>T (rs423904)	C/C	86.2	87.1	N.S.
		T/C	11.7	12.9	N.S.
		T/T	2.1	0.0	N.S.
	+9589A>T (rs454078)	A/A	86.7	85.7	N.S.
		T/A	11.1	14.3	N.S.
		T/T	2.2	0.0	N.S.
	+11100T>C (rs315952)	C/C	34.7	34.3	N.S.
		T/C	46.3	48.6	N.S.
		T/T	19.0	17.1	N.S.
	VNTR	1/1	81.5	83.6	N.S.
		1/2	13.7	10.0	N.S.
		2/2	1.5	0.0	N.S.
		3/1	2.6	2.7	N.S.
		3/3	0.0	0.9	N.S.
		4/1	0.7	2.7	N.S.

N.S., not significant.

Of the four SNPs in *IL1B*, the rs1143627 (−31T>C) and rs16944 (−511C>T) positions in the promoter region of *IL1B* were significantly different between the patient and control groups. For rs16944 (−511 T>C), the frequency of the *C/*C genotype was higher in the patients (34.8%) than in control subjects (21.4%; p=0.025, OR=1.96, 95% CI 0.96<3.96). The *C allele frequency at rs16944 was also higher in the patients (56.5%) than in the control subjects (47.1%; p=0.022, OR=1.46, 95% CI 0.94<2.27). The distribution of rs1143627 (−31C>T) *T/*T, *T/*C, and *C/*C genotype frequencies were 34.4, 41.9, and 23.7, respectively, in keratoconus patients and 21.4, 50.0, and 28.6, respectively, in control subjects. The *T/*T genotype frequency had a higher risk of occurring in keratoconus patients than in control subjects (p=0.027, OR=1.92, 95% CI 0.95<3.90). The allele frequency of *T was 55.4% in keratoconus patients and 46.4% in control subjects (p=0.025, OR=1.43, 95% CI 0.92<2.22).

Of the three SNPs in *IL1A*, the *C/*A genotype for rs2071376 in intron 6 was significantly different between keratoconus patients and control subjects (p=0.034, OR=0.59, 95% CI 0.32<1.11), but the allele frequencies were not significantly different between the groups. Other SNPs and the VNTR showed no significant genotype or allele frequency differences between keratoconus patients and control subjects.

**Figure 2:**
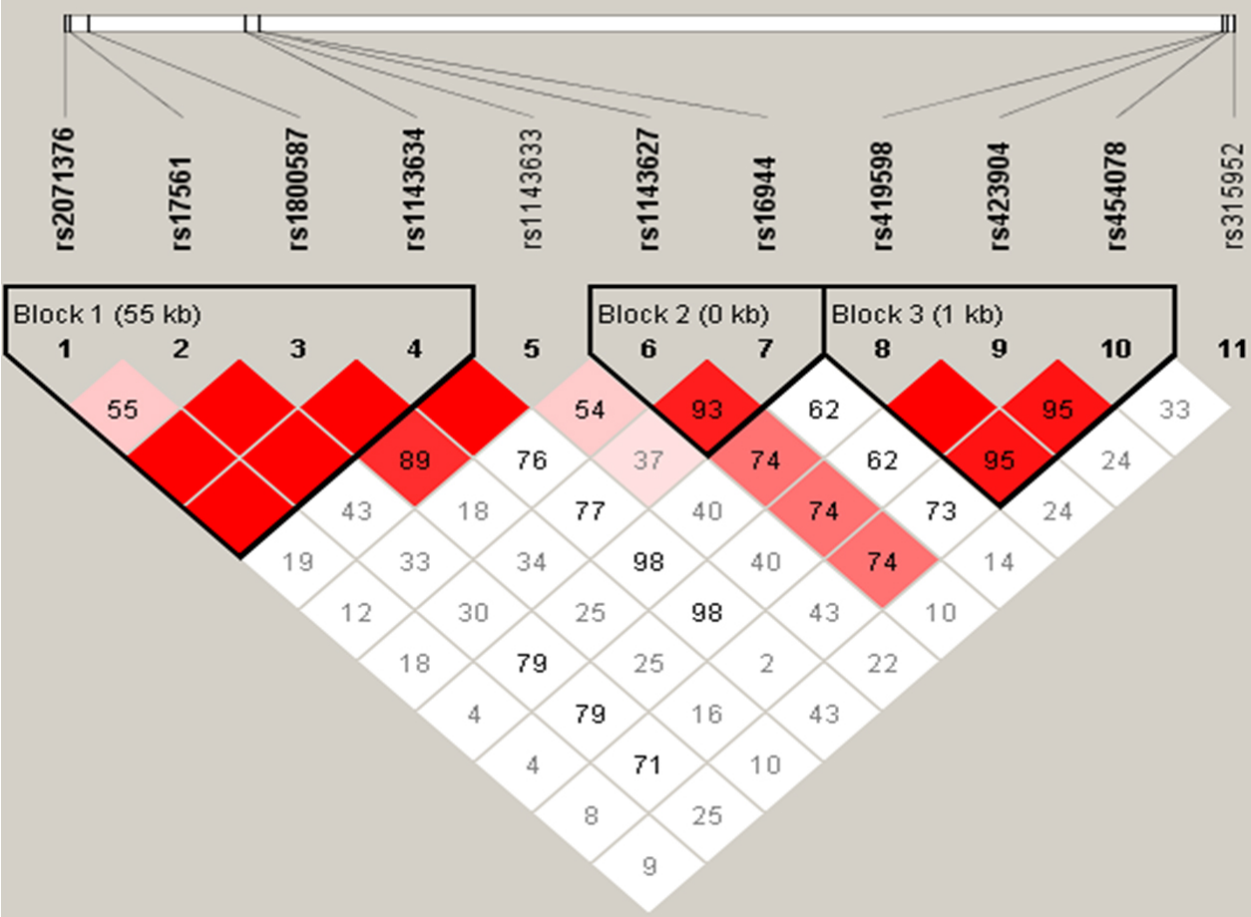
Haplotype structure of select SNPs in *IL1A*, *IL1B*, and *IL1RN*. We estimated the pairwise linkage disequilibrium (LD) by calculating pairwise D' and r^2^ based on marker-marker D'>0.70 and r^2^>0.80. The images were generated with the Haploview software pack.

In the haplotype analysis, we identified three haplotype block sets, rs2071376-rs17561-rs1800587-rs1143634 for Block A, rs1143627-rs16944 for Block B, and rs419598-rs423904-rs454078 for Block C (Figure 2). The T-C haplotype in Block B was more prevalent in keratoconus patients than in control subjects and was associated with a higher risk of developing keratoconus (p=0.034, OR=1.58, 95% CI 1.018<2.448). In contrast, the T-T haplotype in Block B was less frequent in the keratoconus group compared with the control group (p=0.009; Table 4).

**Table 4:** Haplotype analysis of the interleukin 1 genes in keratoconus patients.

**Block**	**Haplotype**	**Case:Control frequency**	**χ^2^**	**p value**
Block 1	rs2071376 -rs17561-rs1800587-rs1143634			
	CGCC	0.656:0.634	0.168	0.682
	AGCC	0.217:0.266	1.031	0.309
	ATTT	0.042:0.029	0.421	0.516
	CTCC	0.041:0.030	0.281	0.596
	ATTC	0.032:0.036	0.043	0.836
Block 2	rs1143627 -rs16944			
	TC	0.556:0.442	4.161	0.034*
	CT	0.394:0.492	3.176	0.075
	CC	0.049:0.029	0.813	0.367
	TT	0.001:0.037	6.168	0.009**
Block 3	rs419598 -rs423904-rs454078			
	TCA	0.916:0.929	0.181	0.67
	CTT	0.074:0.064	0.11	0.74

The asterisk indicates that OR=1.58 and 95% CI=1.018<2.448, and the double asterisk indicates that OR=0.04 and 95% CI=0.003<0.667.

## Discussion

Keratoconus is an abnormality of the interactive epithelial-stromal system that causes a shift in the delicate balance between keratocyte proliferation and apoptosis [12]. Using the TUNEL assay, Kim et al. [12] confirmed the presence of keratocyte apoptosis in keratoconus corneas and the absence of keratocyte apoptosis in normal corneas. Keratocyte apoptosis may be triggered by increased basal IL1 release [12,14,27]. Previous studies indicated that corneal fibroblasts from keratoconus patients express about three times more *IL1A* mRNA than those from a normal cornea [15,28]. Zhou et al. [29] reported that the IL1 protein level was higher in the epithelium and endothelium of the keratoconus corneas than in normal corneas. In addition, IL1 upregulates keratocyte expression of collagenases, metalloproteinases, and other enzymes [30,31]. These enzymes have important roles in the remodeling of collagen during corneal wound healing. For example, persistent rubbing of the eye is likely to produce corneal epithelial trauma and increase the release of IL1 [15,16,32,33]. This would provide a unifying explanation for the association between keratoconus and factors associated with mechanical corneal epithelial cell injury such as poorly fitted rigid contact lenses, excessive eye rubbing, and allergic ocular surface disease [2,4,5,34]. Therefore, it has been suggested that interleukins are central modulators of the response to corneal injury.

The IL1 superfamily includes three secreted glycoproteins, IL1α, IL1β, and IL1Ra. Two of these, IL1α and IL1β, are biologically active while the third member, IL1Ra, is a receptor antagonist that modulates the effects of IL1α and IL1β [18,19]. IL1α and IL1β proteins are synthesized by a variety of cell types including activated macrophages, stimulated B lymphocytes, fibroblasts, and endothelial cells. They are powerful mediators of inflammation and the immune response. *IL1A*, *IL1B*, and *IL1RN* map to human chromosome 2q14. Gene variations that alter gene function are more likely to influence phenotypic characteristics such as risk of disease [18]. An in vivo study showed that the *IL1A*-889 *T allele was associated with increased IL1α and IL1β protein levels. Furthermore, ex vivo analysis of lipopolysaccharide (LPS)-stimulated peripheral blood mononuclear cells indicated that production of IL1α from *IL1A* with the *IL1A*-889*T allele increased [35,36]. The two promoter variants of *IL1B*, −511C>T and −31T>C SNPs, have been repeatedly associated with multiple clinical conditions [37,38] such as cardiovascular disease [39] and gastric cancer [40] as well as with clinically observed differences in the levels of IL1β protein in vivo [39,41]. The *T allele at −31 in the TATA box of the *IL1B* promoter region is suspected to enhance gene expression and induction of IL1β [42]. The -31 *C allele was associated with increased IL1β levels and decreased IL1α levels [43]. It has been reported that the T and C variants at the *IL1B* −31 position regulate gene expression and differential binding of proteins. A specific haplotype, which is composed of the T allele at −511 and the C allele at −31, was significantly associated with a twofold to threefold increase in LPS-induced IL1β protein secretion [21]. There was also nearly a complete linkage disequilibrium between −511C>T and −31T>C in *IL1B* based on a *cis* interaction [40,42].

Some studies have suggested that the allelic polymorphism located within intron 2 of *IL1RN* play a role in differential modulating IL1 activity. The IL1RA*2 is associated with increased IL1Ra protein production in vitro [44]. The *C allele of +8006 C/T in exon 2 of *IL1RN* is associated with the VNTR allele 2 and is associated with lower expression of IL1Ra [45,46]. The *IL1B*-511C, *IL1B*-31T, and *ILRN*+8006T haplotype (C-T-T), which has been associated with lower levels of IL1β expression [21,38], was present in the extended protective haplotype. This extended protective haplotype contains the *IL1RN*+8006T allele, which is associated with elevated IL1Ra expression [45]. Therefore, these studies suggest that *IL1* gene cluster polymorphisms may affect susceptibility to the development of specific diseases and that linkage disequilibrium of functional polymorphisms may indicate increased risk of developing these diseases.

In the present study, we have reported the results of a mutation screening of interleukin 1 genes in unrelated Korean patients with keratoconus. In the screen of four SNPs of *IL1B*, the genotypes of –511 *C/*C and –31 *T/*T of *IL1B* SNPs were associated with a 1.96 fold and 1.92 fold greater risk of developing keratoconus, respectively, (p=0.025 and p=0.027, respectively), showing a strong statistical association with keratoconus in unrelated patients. Two *IL1B* SNPs, −31C/T and −511T/C, have been implicated as potential risk factors for keratoconus among unrelated patients. Of the three SNPs in *IL1A*, the *C/*A genotype of rs2071376 in intron 6 differed significantly between the keratoconus patients and control subjects (p=0.034, OR=0.59, 95% CI 0.32<1.11), but the allele frequencies were not significantly different between the groups.

The T-C haplotype of *IL1B* −31 and –511 was more prevalent in keratoconus patients than in control subjects and carried a higher risk of keratoconus (p=0.034, OR=1.58, 95% CI 1.018<2.448). The T-T haplotype of *IL1B* −31 and −511 was less frequent in keratoconus patients than in the control individuals (p=0.009), which was associated with protection.

Our study is the first to report on *IL1* gene cluster polymorphisms in Korean keratoconus patients. We detected two disease-associated SNPs, rs1143627 (−31T>C) and rs16944 (−511C>T), in *IL1B* and a disease- associated haplotype, the *IL1B*-31T/-511C haplotype, which is associated with regulation of IL1α and IL1β production. It proves that IL1 may have important roles as central modulators of keratoconus development. In conclusion, the present study suggests that two SNPs in *IL1B* predict keratoconus predisposition in unrelated Korean patients, although further research is necessary to elucidate the relationship between the expression levels of IL1α, IL1β, and IL1Ra and SNPs of *IL1B* in keratoconus patients.
